# How to Blister Quickly

**Published:** 1886-01-20

**Authors:** 


**How to Blister Quickly.**—Put a few drops of the concentrated water of ammonia (aqua ammoniæ fort.) in a watch-glass, butter-dish, shallow cup, or other article of like nature, and cover with a pledget of cotton. This inverted and pressed closely to the spot to be blistered, will accomplish this object in from 30 to 60 seconds. It should afterwards be treated as if produced by cantharides.—*Southern Clinic.*

